# Cardiac Tumors: Review

**DOI:** 10.21470/1678-9741-2023-0405

**Published:** 2024-07-15

**Authors:** Carlos J. T. Karigyo, Beatriz Mella S. Pessoa, Samuel Pissinati Nicacio, Emma Terwilliger, Philippos Costa, Pedro Reck dos Santos, Vinicius Ernani, Mahesh Seetharam, Alexandre Noburu Murakami, Felipe Batalini

**Affiliations:** 1 Engineering Center for Circulatory Assistance, Instituto Dante Pazzanese de Cardiologia, São Paulo, São Paulo, Brazil.; 2 Postgraduate Program in Medicine/Technology and Intervention in Cardiology, Universidade de São Paulo, São Paulo, São Paulo, Brazil.; 3 Faculdade de Medicina, Universidade Federal do Amazonas, Manaus, Amazonas, Brazil.; 4 Faculdade de Medicina, Universidade Estadual de Londrina, Londrina, Paraná, Brazil.; 5 Mayo Clinic College of Medicine and Science, Rochester, Minnesota, United States of America.; 6 Division of Hematology and Oncology, Yale University Yale Cancer Center, New Haven, Connecticut, United States of America.; 7 Division of Cardiothoracic Surgery, Mayo Clinic Arizona, Phoenix, Arizona, United States of America.; 8 Division of Oncology, Mayo Clinic Arizona, Phoenix, Arizona, United States of America.; 9 Division of Cardiac Surgery, Universidade Estadual de Londrina, Londrina, Paraná, Brazil.

**Keywords:** Heart Neo plasms, Prognosis, Sarcoma, Lymphoma

## Abstract

Cardiac tumors are rare and encompass a variety of presentations. Clinica symptoms are usually nonspecific, but they can present as obstructive, embolic, or constitutional symptoms. Treatment options and prognosis vary highly depending on the subtype, tumor size, and location. Surgical resection is usually the first-line therapy, except for cardiac lymphomas, and provides favorable long-term prognosis in most benign tumors. Cardiac sarcomas, however, are usually diagnosed in advanced stages, and the treatment relies on a multimodal approach with chemotherapy and radiotherapy. Metastatic cardiac tumors are usually related to advanced disease and carry an overall poor prognosis.

## INTRODUCTION

Primary cardiac tumors are rare neoplasms originating from cardiac tissue. The prevalence of primary cardiac tumors in autopsy studies is about 0.02%, with benign tumors representing 75% of cases, and malignant tumors accounting for 25% of cases. Secondary (metastatic) cardiac tumors are much more common, however, they are clinically silent and tend to be diagnosed postmortem^[1,2]^.

The majority of cardiac tumors are asymptomatic and found incidentally. When present, symptoms are nonspecific and relate to the location of the tumor. Frequent presentations include: 1. systemic or constitutional symptoms; 2. cardiac manifestations, including mass or obstructive effects of blood flow, arrhythmias, pericardial effusion, dyspnea, chest discomfort, or syncope; 3. embolic phenomena — emboli can result from the tumor or nearby thrombi^[3]^.

Intracardiac masses are usually identified using multimodal noninvasive imaging techniques. Advances in cardiac imaging have improved both diagnostic and prognostic rates^[4]^. Treatment is highly variable depending on the tumor histologic type, metastatic spread, clinical presentation, prognosis, and most importantly, the extent of surgical resection^[5]^. Undergoing surgery at a center with relatively higher annual cardiac tumor case load is a predictor of improved survival for patients with cardiac tumors^[6]^.

## IMAGING OF CARDIAC TUMORS

Transthoracic echocardiography (TTE) is generally the initial diagnostic tool due to its wide availability and high resolution to identify small, mobile masses, and its ability to assess intracardiac flow via Doppler echocardiography. However, it is usually incapable of determining the full extent and origin of a mass and has a low capacity for tissue characterization. Transesophageal echocardiography provides a closer evaluation, especially for left-sided structures, and it is routinely conducted after TTE. Contrast echocardiography can also be helpful in distinguishing a tumor from a thrombus^[5,7]^.

Cardiac computed tomography (CT) is the preferred modality for evaluating patients with suspected cardiac metastasis (CMEs) due to its high spatial resolution when assessing the heart and surrounding structures^[5]^. Cardiac CT can also characterize tissues based on different tissue attenuation and radiodensity^[5]^.

Cardiac magnetic resonance (CMR) is the most comprehensive test for identifying and diagnosing cardiac masses due to its ability to identify full anatomic location and extent of the tumor, assess mass mobility, perform functional assessments, and highlight differential tissue properties^[5]^.

Positron emission tomography (PET) evaluates the extent of 18F-fluoro-D-glucose (or FDG) uptake in the tissues to differentiate between benign and malignant tumors. The limitations to PET scans are low sensitivity^[5]^. Although combining new imaging methods, such as CMR, PET, and CT, may increase the sensitivity and specificity of detection, histological examination is still needed to confirm a diagnosis. In this sense, the primary guide to treatment and prognosis is a biopsy of the cardiac lesion^[8]^.

## PRIMARY BENIGN TUMORS

### Myxomas

Cardiac myxomas (CMs) are the most common type of primary cardiac tumor. They most frequently affect women in the age range of 30-60 years. CMs often arise from the interatrial septum of the left atrium at the fossa ovalis. They are pathologically polypoid and round/oval with a smooth or gently lobulated appearance and are histologically derived from proliferating primitive cells that differentiate along endothelial/endocardial lines^[8,9]^. On immunohistochemistry, the neoplastic cells are positive for vimentin, calretinin, S100, nonspecific enolase, factor VIII, CD31, and CD34^[10]^.

It was initially thought that myxomas arose from Prichard structures (endocardial deformities located in the fossa ovalis), however further studies showed no relationship between the two structures. Although not yet clarified, the neural crest was also suggested as a hypothetical origin of CMs since they both express calretinin. Other possible precursors of CM cells include subendothelial vasoformative reservoir cells or cardiomyocyte progenitors^[11]^.

CMs generally occur as an isolated finding, however, < 10% are associated with Carney complex syndrome (CCS). CCS is an autosomal dominant genetic disorder characterized by CMs, spotty skin pigmentation, and endocrine-secreting tumors. More than 70% of patients with CCS exhibit mutations of the PRKAR1A gene at CNC 1 locus. CMs associated with CCS tend to present at a younger age, affect multiple heart chambers, and have a higher chance of recurrence; with women showing the highest probability of recurrence. Yet, the first symptoms tend to occur earlier in men^[12,13]^.

Clinical manifestations vary and are related to the size and location of the tumor. Symptoms of CMs can be related to obstruction (dyspnea, heart murmurs, chest pain), systemic embolization (stroke, myocardial infarction, peripheral embolization), or can be nonspecific (weight loss, fever, fatigue)^[14,15]^.

Tumor detection is usually done through imaging. In an echocardiogram, myxomas appear as spherical masses attached to the endocardial surface, with areas of calcification typically appearing echogenic. In cardiac CT and magnetic resonance imaging (MRI), the tumors often appear heterogeneous and with different shapes. The tumors may also enhance after contrast administration, especially on MRI^[9]^.

CMs and thrombi are occasionally difficult to distinguish from one another, even though they are managed in a completely different manner. The radiomic signature based on cardiovascular contrast-enhanced CT can help improve diagnostic efficiency. Other differential diagnoses include lipomatous hyperplasia of the interatrial septum, lipomas, inflammatory diseases, and metastasis to the heart^[16,17]^.

**Fig. 1:**
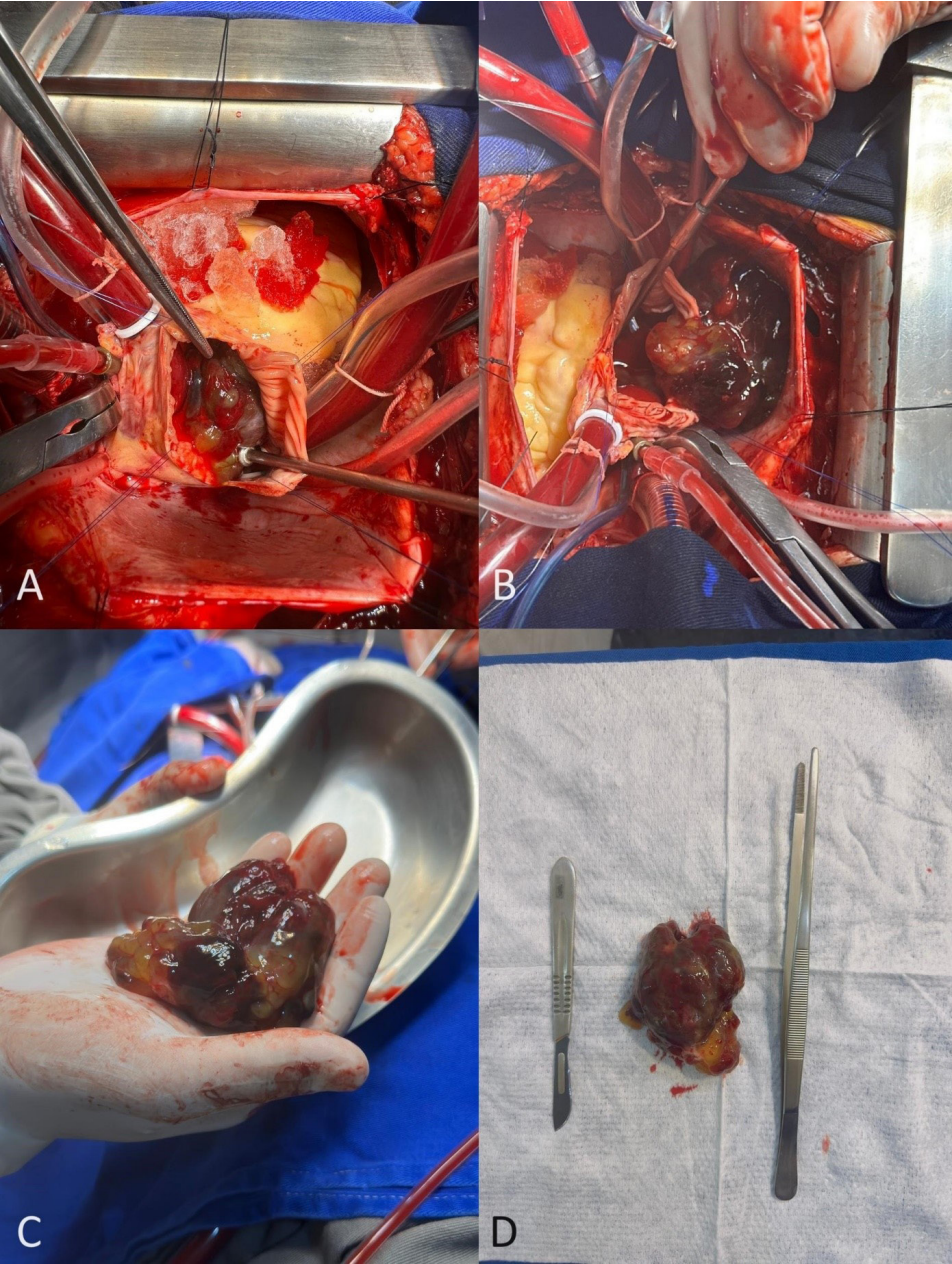
Resection of a giant right atrial myxoma, which caused right ventricular outflow tract obstruction. (A) Intraoperative view of the myxoma located inside the right atrium. (B) Tumor resection required the excision of a part of the right atrial wall. (C) and (D) Excised giant myxoma from the right atrium.

**Fig. 2:**
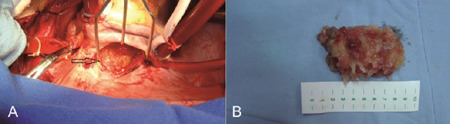
Resection of a left atrial myxoma, which caused mitral valve obstruction. (A) Intraoperative view of a myxoma located in the left atrium (arrow). (B) The myxoma after excision.

Surgical excision is the first line treatment for CMs (Figures 1 and 2) and usually provides long-term survival in patients. There are different surgical approaches depending on the tumor’s location and characteristics^[18]^. Myxomas have been successfully excised using robotic technology, with advantages being a shorter hospital length of stay, reduced postoperative pain, and a more rapid functional recovery^[19]^.

### Rhabdomyomas

Cardiac rhabdomyoma (CR) is the most common cardiac tumor in children, frequently detected in early prenatal ultrasounds, with the potential to involve the myocardium of both ventricles and the interventricular septum^[20]^. They are occasionally associated with tuberous sclerosis complex (TSC), a genetic disorder caused by a mutation in two tumor suppressor genes — TSC1 (hamartin) and TSC2 (tuberin)^[21]^. The classic triad of symptoms of TSC include seizures, intellectual disability, and facial angiofibromas. However, clinical manifestations can be highly variable and involve multiple systems, such as dermatologic, ophthalmic, and renal^[22]^. CRs are usually asymptomatic and tend to regress after birth, but can present with fetal arrhythmias, nonimmune hydrops fetalis, respiratory distress, congestive heart failure, or cyanosis^[20]^.

In histology, tumor cells with a spider-like morphology (“spider cells”) are the classical finding for CRs^[20]^. In terms of immunohistochemistry, CR tumor tissue expresses autophagic proteins (P62 and LC3b) and apoptotic proteins (caspases 3 and 7). The findings of autophagy and apoptosis may be related to tumorigenesis and regression of the tumor^[23]^.

Asymptomatic patients tend to be managed conservatively with close follow-up exams and serial echocardiography, since most CRs regress spontaneously. In patients with ventricular outflow obstruction or signs of severe hemodynamic compromise, surgical intervention is an option^[24]^.

Overactivity of the mammalian target of rapamycin (mTOR) pathway is believed to be related to the pathophysiology of TSC. In this context, mTOR inhibitors (mTORi), such as sirolimus and everolimus, have emerged as new therapeutic options in the management of TSC manifestations. Some case reports have found significant CRs size reduction and clinical improvement with mTORi therapy^[25,26]^. However, with the natural tendency for TSC to regress with age, the reduction of CRs size may not have resulted exclusively from medical therapy, and the favorable findings could reflect the natural clinical course of the tumor.

A systematic review has shown that mTORi treatment effectiveness and safety are insufficient to recommend this therapy universally for all CRs. However, it can be considered as a temporary life-saving therapeutic option, especially for symptomatic, large CRs or when the risk of surgical intervention is significant^[27]^. The ORACLE (everOlimus for caRdiac rhAbdomyomas in tuberous sCLErosis) trial is an ongoing, phase II, randomized clinical trial assessing the efficacy of everolimus as a specific therapy for CR. It involves 40 children with symptomatic CR secondary to TSC. The trial results will potentially be the first evidence-based therapy for this condition^[28]^.

### Papillary Fibroelastoma

Papillary fibroelastomas usually present as a small, round pedunculated valvular/endocardial mass with multiple papillary projections. Their gross pathological appearance is compared to that of a sea anemone^[29]^. The aortic valve is the most frequently affected location, with the pulmonary valve being the least affected. Although they have benign histology and are usually asymptomatic, they have the potential for embolic events, such as transient ischemic attack, stroke, myocardial infarction, syncope, and pulmonary and peripheral embolism. Therefore, the standard of care is surgical excision^[30]^. Papillary fibroelastoma is the most frequently excised heart tumor, nearly twice as frequent as CMs. This has sparked a debate on its potential neoplastic nature^[8]^.

### Fibromas

Cardiac fibromas (CF) are solitary masses that occur primarily in the free wall of the left ventricle and the ventricular septum, commonly affecting infants and children. CF can also be the initial manifestation of an autosomal dominant disease known as nevoid basal cell carcinoma syndrome (Gorlin-Goltz syndrome)^[31]^.

Histologically, CFs are composed of fibroblasts and connective tissues. Infants and young children tend to exhibit more inflammatory infiltration than adults and have a higher chance of receiving the wrong diagnosis of an aggressive or malignant lesion^[31]^. In echocardiography, they often show increased echogenicity in contrast to normal myocardium^[32]^.

Clinical presentations of CFs can include symptoms of heart failure, arrhythmias, dyspnea, and chest pain. Surgical resection is the recommended treatment since the tumor has the potential to infiltrate the surrounding myocardium and act as a substrate for ventricular arrhythmias^[31]^.

### Lipomas

Lipomas are masses derived from mature adipocytes enclosed by a collagenous capsule, frequently involving the endocardium of the right atrium, left ventricle, and the pericardium. Risk factors include a high body mass index, older age, and female sex. Symptoms depend on the size and location of the tumor. However, lipomas are mainly asymptomatic. Embolic events are rare since the mass is encapsulated. Differential diagnosis includes liposarcomas and lipomatous hypertrophy of the interatrial septum. Surgery is usually indicated for large lipomas and symptomatic patients^[33,34,35,36]^.

### Hemangiomas

Cardiac hemangiomas (CH) are benign tumors characterized by the proliferation of endothelial cells, leading to an increase in vascularization. Microscopic types include cavernous (the most frequent subtype), capillary, and arteriovenous. They can originate from the pericardium, myocardium, or endocardium; the most common location is the right atrium, although the septum (interatrial and interventricular) can also be affected. The natural history of CH is unpredictable, but most patients remain stable, and the tumor can regress spontaneously. Despite its benign histopathology, CH carries the risk of life-threatening complications like syncope and stroke. Surgical excision is usually the first-line therapy, but biopsy alone is not indicated due to adverse effects. Options for medical therapy include vascular endothelial growth factor (VEGF) antagonists, beta-blockers, and corticosteroids^[37]^.

## PRIMARY MALIGNANT TUMORS

### Angiosarcomas

Angiosarcoma (AS) is the most common type of cardiac sarcoma, mostly diagnosed in middle-aged men, and has the worst prognosis compared to other malignant tumors affecting the heart^[38]^. In a retrospective analysis of 10 patients with AS, the overall median survival was 5.2 months, while the estimated one-year survival rate was 35%^[39]^.

AS demonstrates a predilection for the right atrium and has the potential to invade surrounding structures, such as the pericardium, inferior vena cava, and tricuspid valve. The most frequent sites of metastasis are lungs and bones, but spleen and liver can also be affected^[38]^. Patients can also develop brain metastases, particularly associated with left heart disease, and some specialists recommend brain imaging at the time of diagnosis^[40]^. A significant risk factor for AS includes radiation^[41]^. Clinical manifestations include chest pain, dyspnea, arrhythmias, and malignant pericardial/pleural effusions^[38]^.

AS is characterized by abnormal, pleomorphic, malignant endothelial cells. These cells can appear rounded, polygonal, or fusiform, and may even exhibit an epithelioid appearance^[42]^. Immunological staining of AS is typically positive for endothelial markers such as von Willebrand factor, CD31, and CD34. Most notably, ERG has shown high sensitivity to AS, so its use should be considered when investigating cardiac AS^[39]^. Molecular aberrations include KDR, KIT, CDKN2A, MYC, ARID1A, and TP53^[41,43]^. Surgery is the preferred treatment. However, due to local invasion and frequent metastases by the time of diagnosis, full resection is often not feasible^[38,41,43]^. In this setting, neoadjuvant therapy has been shown to increase the rate of resectability (R0 resection), also leading to improved survival^[44]^. Additionally, adjuvant treatments are controversial, but chemotherapy and radiotherapy are often used as part of a multimodal approach^[39,41]^.

Multiple systemic treatments are employed in AS^[43]^. Doxorubicin-based regimens, typical for soft tissue sarcomas, can be used for AS, but their use for patients with heart failure is limited due to cardiotoxicity^[43]^. A retrospective study analyzed adjuvant doxorubicin-containing chemotherapy in 15 healthy patients with primary cardiac sarcoma (PCS) (six of which had AS). The median interval to first relapse was 10 months in PCS *vs.* 3.5 months in AS, and the median survival was 12 months in PCS *vs.* six months in AS^[45]^. In another case report, a patient with primary cardiac AS survived three years following surgical resection and chemoradiation^[44,46]^. Taxanes have shown activity in AS^[47]^. A patient who underwent surgical resection of a cardiac AS followed by adjuvant docetaxel and radiotherapy achieved an overall survival (OS) time of 32 months^[48]^. Three other patients diagnosed with AS after presenting with circulatory collapse recovered from hemodynamic instability following the initiation of weekly paclitaxel chemotherapy and showed a partial response after six months^[46]^. Targeted therapies have also been used in the management of AS^[49]^. In another case involving recurrent cardiac AS, aggressive surgical resection combined with pazopanib, a multi-targeted tyrosine kinase inhibitor against VEGF receptor (VEGFR), led to a patient survival time of two years with complete remission of disease^[50]^. Immunotherapy has also shown activity in AS, including cardiac AS^[51-53]^. A study on 14 patients with PCS (of which 43% were AS) treated with immune checkpoint inhibitors showed an overall response rate of 35.6 months^[54]^.

Cardiac AS often presents with mutations on KDR and KIT and homozygous deletion of CDKN2A. KDR is a kinase receptor that encodes for one of the VEGFR tyrosine kinases; its relation to angiogenic signaling pathways provides a rationale for targeted therapies^[43]^. The multi-targeted tyrosine kinase inhibitor, pazopanib, was approved for metastatic soft-tissue sarcoma, after the results of the phase 3 study PALETTE showed a median progression-free survival of 4.6 months with 1.6 months for placebo^[55]^. Pazopanib represents a valuable therapeutic option in cardiac AS, and some case reports have shown improved patient survival, especially when associated with surgical resection^[50,56]^.

### Rhabdomyosarcomas

Rhabdomyosarcomas (RMS) arise from undifferentiated skeletal tissue and are the second most common type of malignant cardiac tumor, accounting for 21% of PCS. They frequently infiltrate the pericardium and cardiac valves, interfering with valvular function^[57]^. This tumor may also be associated with radiation therapy^[58]^. The prognosis of cardiac RMS is poor, with a post-diagnosis and surgical resection survival time being < 1 year. Multimodality treatment with chemotherapy, surgery, and radiation is the mainstay of treatment for RMS, which can be used in the curative and palliative setting. A commonly used chemotherapy regimen for RMS consists of vincristine, actinomycin D, and cyclophosphamide (VAC)^[59,60,61,62]^. Alternative regimens include vincristine, actinomycin D, and ifosfamide (or VAI), vincristine, ifosfamide, and etoposide (or VIE), or vincristine, doxorubicin, and cyclophosphamide, in addition to ifosfamide/etoposide (or VDC/IE)^[63]^. There was also a successful case of primary cardiac RMS treated with eribulin as a second line agent after a trial of VAC regimen^[62]^.

### Leiomyosarcoma

Leiomyosarcoma (LMS) is a form of soft tissue sarcoma composed of cells with distinct smooth muscle features most commonly located at the left atrium. LMS immunohistochemistry is commonly positive for desmin and smooth muscle actin (smooth muscle markers). In imaging studies, the tumor can sometimes look like a CM. Clinical presentations include dyspnea, chest discomfort, and arrhythmia-related symptoms. Prognostics are still unclear, and the most common treatment modality is surgery^[8]^. Adjunctive chemotherapy and radiotherapy are also used. A review of LMS documented a median survival time of 14 months^[64]^.

### Undifferentiated Pleomorphic Sarcoma

Undifferentiated pleomorphic sarcoma (UPS) is a high-grade sarcoma with a poor prognosis and a high variable morphology that frequently shows nuclear pleomorphism with spindle shaped cells. UPS can be difficult to differentiate from myxofibrosarcoma due similar morphology^[65]^. These tumors are often negative for specific markers of muscular lineage; however, some UPS can present with an MDM2 amplification on fluorescence *in situ* hybridization molecular analysis^[66,67]^.

### Osteosarcoma

Primary cardiac osteosarcoma is extremely rare, predominantly observed in the left atrium. Other subtypes of conventional osteosarcoma that arise in the bone, such as osteoblastic, chondroblastic, and fibroblastic types, can also be seen in primary cardiac osteosarcomas. The most common metastatic sites are the brain and bones^[68]^. A review of previous cases showed a five-year OS and disease-free survival rate of 33.5% and 6.3%, respectively. The median OS of the patients diagnosed antemortem was approximately 20 months^[65]^.

### Synovial Sarcoma

Synovial sarcoma accounts for < 5% of all PCS. It appears most commonly in men and can involve the pericardium and right ventricular outflow tract. Grossly, the tumors are large and covered with fibrous pseudocapsules. Imaging studies often reveal large pericardial or intracardiac/endocavitary masses. However, these findings are not characteristic of synovial sarcoma, and a diagnosis relies on histopathology and identification of its pathognomonic translocation, t(X;18)(p11;q11). It can also be distinguished from cardiac AS due to the lack of expression of ERG and CD34^[69]^.

### Fibrosarcomas

Cardiac fibrosarcoma is a rare malignant tumor of mesenchymal origin. Symptoms are nonspecific and relate to the anatomical location^[70]^. Surgery is the primary treatment. Cardiac transplantation also remains an option for patients with localized disease and nonresectable tumors, with intermediate/long-term survival sometimes being achieved. Adjuvant chemotherapy and radiotherapy can also help with symptom relief and prolong survival^[71]^. Infantile fibrosarcomas show neurotrophic tyrosine receptor kinase fusion in > 90% of the cases, and this mutation can be targeted as a treatment option through the use of tropomyosin receptor kinase inhibitors^[72]^.

### Lymphomas

Primary cardiac lymphoma is a rare type of non-Hodgkin lymphoma that involves the heart and/or pericardium. It most commonly affects the right atrium, although any chamber may be involved. It is associated with Epstein-Barr virus infections, immunosuppression, and chronic inflammation. Dyspnea is the most common presenting symptom, followed by constitutional complaints and chest pain. Other clinical presentations can include signs of congestive heart failure, arrhythmias such as atrioventricular blocks, pericardial effusion, or masses, cardiac tamponade, and superior vena cava syndrome. In terms of histological subtype, diffuse large B-cell lymphoma (DLBCL) is the most common^[8,73]^. Common metastatic involvement can include the central nervous system, testicle, kidney, adrenal gland, skin, and breast^[74]^.

Lymphomas can mimic other neoplasms such as cardiac sarcomas and metastatic bronchogenic carcinomas on imaging studies; therefore, histopathology remains the gold standard for diagnosis^[75]^.

Treatment options include chemotherapy, surgical resection, and radiotherapy. Chemotherapy remains the most common treatment modality and is associated with improved survival. The regimen of choice is cyclophosphamide, doxorubicin, vincristine, and prednisone (CHOP) or CHOP + rituximab^[76]^. Complete resolution and remission of cardiac lymphoma following chemotherapy has been reported^[77]^ The use of rituximab in a cohort of patients with DLBCL showed an increase in OS, although it was not statistically significant^[78]^. Surgical intervention also remains an option for patients who are hemodynamically unstable, unable to tolerate chemotherapy or with unsuccessful response, or in palliative care^[79]^. Although it may relieve the ventricular outflow obstruction and reduce risk of sudden death, surgical intervention does not seem to improve OS^[74]^.

## SECONDARY/METASTATIC TUMORS

CMEs are significantly more likely to be diagnosed than primary cardiac tumors; however, they are more frequently visualized during autopsy due to nonspecific findings. In an autopsy study involving 1294 adult patients with solid tumors, the incidence of CMEs was 4.71%. CMEs occur in disseminated cancers and indicate advance-stage disease. Frequent sites of primary cancers that metastasize to the heart include lung, pleural mesothelioma, skin melanoma, breast, esophagus, and hematologic malignancies^[5,80]^. Unusual sites of CMEs reported in the literature include uterus, vulva, thyroid, liver, colorectal, renal, and head & neck cancer^[81,82,83,84,85,86,87]^. Secondary cardiac involvement can occur by direct spread to the heart from thoracic malignancies such as tumors involving the lungs, which typically behave by initial invasion of the pulmonary veins and sequential extension to the left atrium, including endoluminal extension. In very selected cases, resection can be possible, and often times including the use of cardiopulmonary bypass and left atrial reconstruction^[88]^. Additionally, hematogenous, transvenous (through inferior vena cava), and lymphatic dissemination can also be causes of cardiac involvement. The most frequent location of CMEs is the pericardium, accounting for two-thirds of cases, followed by the myocardium and/or epicardium (one third of cases), and lastly by the endocardium (5% of the cases)^[2,80]^.

CMEs are associated with changes in the standard 12-lead surface electrocardiography (ECG), notably causing ST segment elevation (STE) with a convex shape. This finding could be related to the replacement of necrotic and/or electrically inactive tissue into the affected region of the myocardium. STE is not accompanied by pathologic Q waves or ECG evolution^[89,90]^.

Table 1 is a summary of the main characteristics of cardiac tumors.

**Table 1:** Summary of the main characteristics of cardiac tumors.

Tumors	Characteristics
**Primary benign**	**Myxoma:** most common type of primary cardiac tumor, 30-60-year-old women, interatrial septum of the left atrium, polypoid and round, surgery is the first line treatment
	**Rhabdomyoma:** most common cardiac tumor in children, involving the myocardium of both ventricles and interventricular septum, may regress spontaneously, surgical treatment
	**Papillary fibroelastoma:** small, round pedunculated valvular mass with multiple projections, most commonly in aortic valve, risk for embolic events, surgical treatment
	**Fibroma:** commonly affecting infants and children, solitary mass, free wall of the left ventricle and ventricular septum, surgical treatment, present in Gorlin-Goltz syndrome
	**Lipoma:** derived from mature adipocytes enclosed by a collagenous capsule, frequently involving the endocardium of right atrium, left ventricle and pericardium, surgical treatment
	**Hemangioma:** proliferation of endothelial cells, leading to an increase in vascularization, most common location in right atrium, surgery usually is the first choice of treatment
**Primary malignant**	**Angiosarcoma:** the most common and has the worst prognosis, mostly diagnosed in middle-aged men, predilection for right atrium, surgical treatment is preferred
	**Rhabdomyosarcoma:** second most common, arising from undifferentiated skeletal tissue, frequently infiltrates pericardium and heart valves, multimodality treatment
	**Leiomyosarcoma:** soft tissue sarcoma, different smooth muscle features, most common in left atrium, may look like a myxoma in imaging studies, surgical treatment
	**Undifferentiated pleomorphic sarcoma:** high-grade sarcoma with a high variable morphology, nuclear pleomorphism with spindle shaped cells
	**Osteosarcoma:** extremely rare, most observed in left atrium, subtypes may be present osteoblastic, chondroblastic, and fibroblastic)
	**Synovial sarcoma:** most common in men and involves pericardium and right ventricular outflow tract, grossly large and covered with fibrous pseudocapsules
	**Fibrosarcoma:** mesenchymal origin, surgical treatment including transplantation, TRK inhibitors in infantile fibrosarcomas
	**Lymphoma:** non-Hodgkin lymphoma, most common in right atrium, CHOP or CHOP + rituximab chemotherapy regimen
**Secondary or metastatic**	Frequent sites of primary cancers that metastasize to the heart include lung, pleural mesothelioma, skin melanoma, breast, esophagus, and hematologic malignancies. The most frequent location is the pericardium, accounting for two-thirds of cases. Surgical resection for selected cases, including the use of cardiopulmonary bypass

CHOP=cyclophosphamide, doxorubicin, vincristine, and prednisone; TRK=tropomyosin receptor kinase

## CONCLUSION

Cardiac tumors are rare and have a variety of presentations, ranging from small benign masses with highly favorable prognosis to sarcomas in advanced stages with limited therapeutic options. Clinical symptoms are usually nonspecific, which can lead to a late diagnosis or be an incidental finding. The symptoms that present are often related to the obstruction and local invasion caused by the tumor, as well as embolic manifestations. Benign primary tumors usually benefit from surgical resection and provide favorable long-term prognosis, but selected asymptomatic patients can also be managed conservatively, depending on tumor subtype. Surgical resection is the first option in cardiac sarcomas. However, due to the potential for local expansion, distant metastasis, and overall poor prognosis, a multimodal therapy involving chemotherapy and radiotherapy is frequently used, despite the concern for adverse effects. Cardiac lymphomas are initially managed with chemotherapy. Metastatic cardiac tumors, when diagnosed, are usually a sign of advanced disease, and carry an overall poor prognosis.

## Abbreviations, Acronyms & Symbols

AS: Angiosarcoma
CCS: Carney complex syndrome
CF: Cardiac fibromas
CH: Cardiac hemangiomas
CHOP: Cyclophosphamide, doxorubicin, vincristine, and prednisone
CMEs: Cardiac metastasis
CMR: Cardiac magnetic resonance
CMs: Cardiac myxomas
CR: Cardiac rhabdomyoma
CT: Computed tomography
DLBCL: Diffuse large B-cell lymphoma
ECG: Electrocardiography
LMS: Leiomyosarcoma
MRI: Magnetic resonance imaging
mTOR: Mammalian target of rapamycin
mTORi: mTOR inhibitors
OS: Overall survival
PCS: Primary cardiac sarcoma
PET: Positron emission tomography
RMS: Rhabdomyosarcomas
STE: ST segment elevation
TRK: Tropomyosin receptor kinase
TSC: Tuberous sclerosis complex
TTE: Transthoracic echocardiography
UPS: Undifferentiated pleomorphic sarcoma
VAC: Vincristine, actinomycin D, and cyclophosphamide
VEGF: Vascular endothelial growth factor
VEGFR: Vascular endothelial growth factor receptor
